# Evaluating the toxicity of TiO_2_-based nanoparticles to Chinese hamster ovary cells and *Escherichia coli:* a complementary experimental and computational approach

**DOI:** 10.3762/bjnano.8.216

**Published:** 2017-10-17

**Authors:** Alicja Mikolajczyk, Natalia Sizochenko, Ewa Mulkiewicz, Anna Malankowska, Michal Nischk, Przemyslaw Jurczak, Seishiro Hirano, Grzegorz Nowaczyk, Adriana Zaleska-Medynska, Jerzy Leszczynski, Agnieszka Gajewicz, Tomasz Puzyn

**Affiliations:** 1 Laboratory of Environmental Chemometrics, Faculty of Chemistry, University of Gdansk, Wita Stwosza 63, 80-308 Gdansk, Poland; 2 Interdisciplinary Center for Nanotoxicity, Jackson State University, 39217, Jackson, MS, USA; 3 Department of Environmental Analytics, University of Gdansk, Wita Stwosza 63, 80-308 Gdansk, Poland; 4 Department of Environmental Technology, University of Gdansk, Wita Stwosza 63, 80-308 Gdansk, Poland; 5 Department of Biomedical Chemistry, University of Gdansk, Wita Stwosza 63, 80-308 Gdansk, Poland; 6 Center for Environmental Risk Research, National Institute for Environmental Studies, Tsukuba, 16-2 Onogawa, Ibaraki 305-8506, Japan; 7 NanoBioMedical Centre, Adam Mickiewicz University, Umultowska 85, 61-614 Poznan, Poland

**Keywords:** Au/Pd–TiO_2_ photocatalyst, bimetallic nanoparticles, nanotoxicity, nano-QSAR, second-generation nanoparticles

## Abstract

Titania-supported palladium, gold and bimetallic nanoparticles (second-generation nanoparticles) demonstrate promising photocatalytic properties. However, due to unusual reactivity, second-generation nanoparticles can be hazardous for living organisms. Considering the ever-growing number of new types of nanoparticles that can potentially contaminate the environment, a determination of their toxicity is extremely important. The main aim of presented study was to investigate the cytotoxic effect of surface modified TiO_2_-based nanoparticles, to model their quantitative nanostructure–toxicity relationships and to reveal the toxicity mechanism. In this context, toxicity tests for surface-modified TiO_2_-based nanoparticles were performed in vitro, using Gram-negative bacteria *Escherichia coli* and Chinese hamster ovary (CHO-K1) cells. The obtained cytotoxicity data were analyzed by means of computational methods (quantitative structure–activity relationships, QSAR approach). Based on a combined experimental and computational approach, predictive models were developed, and relationships between cytotoxicity, size, and specific surface area (Brunauer–Emmett–Teller surface, BET) of nanoparticles were discussed.

## Introduction

Unmodified titania (TiO_2_) nanoparticles (so-called first-generation NPs) represent a material that alters the rate of chemical reactions, when exposed to light (photocatalyst) [1]. TiO_2_-based NPs have already found wide applications as efficient photocatalysts for sterilization, sanitation, air and water purification systems, hydrogen production by water splitting, and dye-sensitized solar cells [1]. Photocatalysis is an effective and environmentally friendly photooxidation process [2]. Development and application of photocatalysis techniques are reasonably economical endeavor. Photocatalysis could be a more reliable method than traditional methods for the inactivation of bacteria (i.e., UV disinfection and chlorination).

Unmodified titania NPs are generally considered to be inert and non-toxic [3]. However, several studies have reported that TiO_2_ nanomaterials may elicit toxic effects towards bacteria under UV light [4], which makes it possible to use them as an antibacterial material [5–6]. In TiO_2_-based nanoparticles, electron/hole (e^−^/h^+^) pairs can be generated under UV light. Under such conditions, free radicals are produced, which is one of the major pathways of the antibacterial activity of TiO_2_-based NPs. In the absence of UV light, photoactive TiO_2_ nanomaterials demonstrate little or no bacteria inhibiting activity [5–6]. Reactivity of TiO_2_ under visible light (λ > 400 nm) can be achieved in several ways [7], including: (a) metal doping [8], (b) non-metal doping [9–10], (c) self-doping (reductive treatments) [11–12], (d) surface modification by noble-metal nanoparticles of silver (Ag), gold (Au), platinum (Pt), or palladium (Pd) [13–14], (e) the use of dye-modified TiO_2_ [15–16], or (f) coupling TiO_2_ with other semiconductors [17–18]. In the current work, we will focus on surface modification methods.

Metal-ion doped TiO_2_ (so-called second generation nanomaterials) may cause adverse effects not only towards bacteria, but also exhibit detrimental effects to the environment and to human health. Many studies have been focused on ways to synthesize doped NPs, leveraging the photocatalytic (UV–vis active photocatalyt) and bactericidal properties, and minimizing the release of potentially toxic ions. For instance, TiO_2_ NPs doped with either copper (Cu) or silver (Ag), exhibited enhanced antibacterial activity against *Staphylococcus aureus*, whereas their toxicity towards mouse cells from L929 cell line remained low [19]. Ag-TiO_2_ NPs, which were activated by UV–vis light, exhibited stronger bactericidal activity (towards Gram-positive *B. subtilis* and Gram-negative *P. putida*) than NPs activated by UV [4]. At the same time, no significant cytotoxicity has been detected for TiO_2_ doped with nitrogen (N), gold (Au) or selenium (Sn) [20–21]. Whereas, copper oxide-doped TiO_2_ and iron/nitrogen co-doped (Fe/N-co-doped) TiO_2_ nanocomposite particles were detectably cytotoxic [22]. More complex nanostructures of TiO_2_ bilayer nanosheets doped with bismuth tungstate (Bi_2_WO_6_) nanoclusters demonstrated enhanced antimicrobial activity towards *E. coli*: the bacteria population continuously decreased with the increasing concentration of Bi_2_WO_6_ [23]. In another contribution, the photo-oxidation capability of iron-doped TiO_2_ NPs increased during exposure to near-visible light. Fe-doped TiO_2_ NPs inhibited the macrophage RAW 264.7 [24].

Hence the same unique properties of surface-modified TiO_2_-based nanomaterials that offer a bunch of new opportunities for the advancement of nanotechnology could result in unknown risks to human health and the environment. Our attention should be focused both on the promise of new opportunities and on the responsibility of industries to guarantee the safety of their products for workers, consumers and the environment. The conventional (i.e., experimental) risk assessment approaches using laboratory animals are often expensive, time-consuming and problematic from an ethical point of view. Thus novel, fast and cheaper procedures for risk assessment are necessary, without the requirement of extensive animal testing. The development of computational methods complimentary to the experiments, and capable of supporting the empirical testing is of increasing interest. This idea has been expressed in the established EU REACH (Registration, Evaluation, Authorization and Restriction of Chemicals) regulations, which pronounce that information about risk assessment of chemicals should be generated whenever possible by means other than vertebrate animal tests, through the use of alternative methods, for example quantitative structure–activity relationship models (QSAR) [25–26]. This approach is based on defining mathematical dependencies between the variance in molecular structures, encoded by so-called molecular descriptors, and the variance in a given physicochemical property or biological (e.g., cytotoxicity) property in a set of compounds (“endpoints”) [5,25–33]. Preliminary studies proved that the development of novel computational methods might significantly reduce the number of required animal experiments [27,33–45].

The aim of our study was to investigate cytotoxic effects of TiO_2_-based second-generation nanoparticles using the combination of experimental and computational techniques. Here, we present the quantitative description of adverse effects of modified titania nanomaterials towards Chinese hamster ovary (CHO-K1) cells and Gram-negative bacteria *Escherichia coli*.

## Results and Discussion

### Toxicity evaluation

Three types of TiO_2_-based NPs were synthetized: (1) monometallic (Au, Pd) clusters, (2) core–shell particles and (3) alloy bimetallic clusters (Au/Pd). The cytotoxicity and antimicrobial activity of TiO_2_ modified with palladium and/or gold NPs is presented in Table 1. Inhibition of bacterial growth was not observed up to the highest tested concentration of 500 µg/L. In the agar diffusion method, a slight inhibition of bacterial growth was noted for nanomaterials: 0.25Au, 1.25Au, 0.1Pd_0.5Au, 0.1Pd_1.25Au, 0.5Pd_0.5Au. At the same time, mammalian cells were more sensitive to Au/Pd-TiO_2_ NPs (Table 1).

**Table 1:** Cytotoxicity and antimicrobial activity (MIC and microorganism growth inhibition zone) of TiO_2_ modified with Pd and/or Au nanoparticles.

sample label	cytotoxicity EC_50_ (µg/mL)	antimicrobial activity	
		MIC (µg/mL)	zone of inhibition diameter (mm)^a^

pure TiO_2_	>300	>500	6
0.1Au	187.50 ± 0.43	>500	6
0.25Au	118.39 ± 2.19	>500	9
1.25Au	156.80 ± 0.42	>500	9
0.1Pd	164.18 ± 1.75	>500	6
0.25Pd	186.35 ± 1.73	>500	6
0.5Pd	204.02 ± 1.96	>500	6
0.5Pd_1.25Au	275.63 ± 3.13	>500	6
0.1Pd_0.1Au	165.96 ± 0.76	>500	6
0.1Pd_0.25Au	158.00 ± 0.92	>500	6
0.1Pd_0.5Au	195.73 ± 1.82	>500	7
0.1Pd_1.25Au	155.35 ± 2.38	>500	7
0.5Pd_0.1Au	141.91 ±1.73	>500	6
0.5Pd_0.25Au	175.12 ± 1.91	>500	6
0.5Pd_0.5Au	162.68 ± 2.29	>500	7
0.25Pd_0.25Au	134.28 ± 1.35	>500	6
0.25Pd_0.5Au	241.73 ± 3.07	>500	6
0.25Pd_1.25Au	220.80 ± 0.51	>500	6

^a^A value of 6 equals the diameter of the paper disc, i.e., no inhibition occurred.

**Figure 1:**
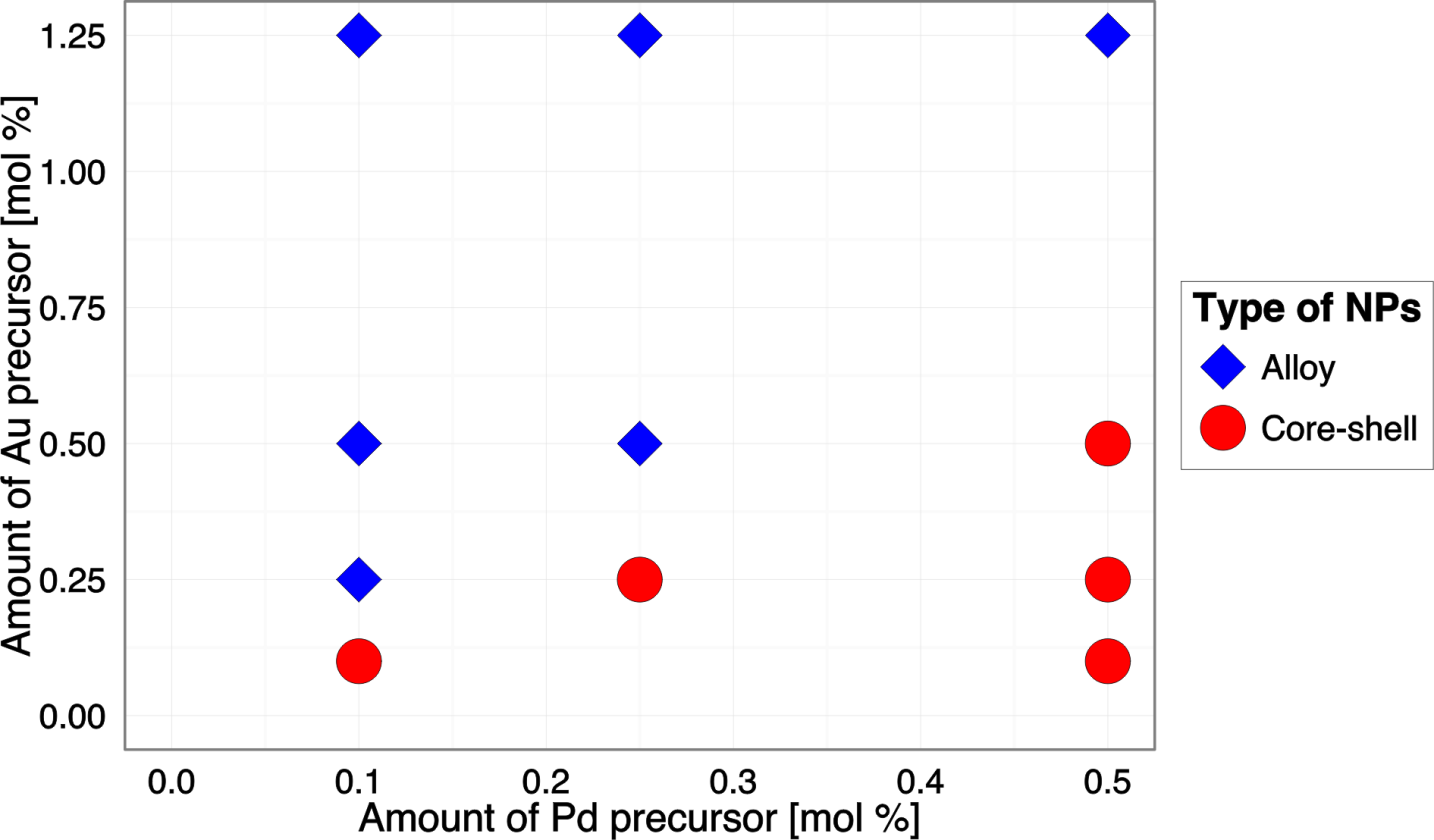
The effect of the amount and type of metal precursor on the structure of the nanoparticles.

**Figure 2:**
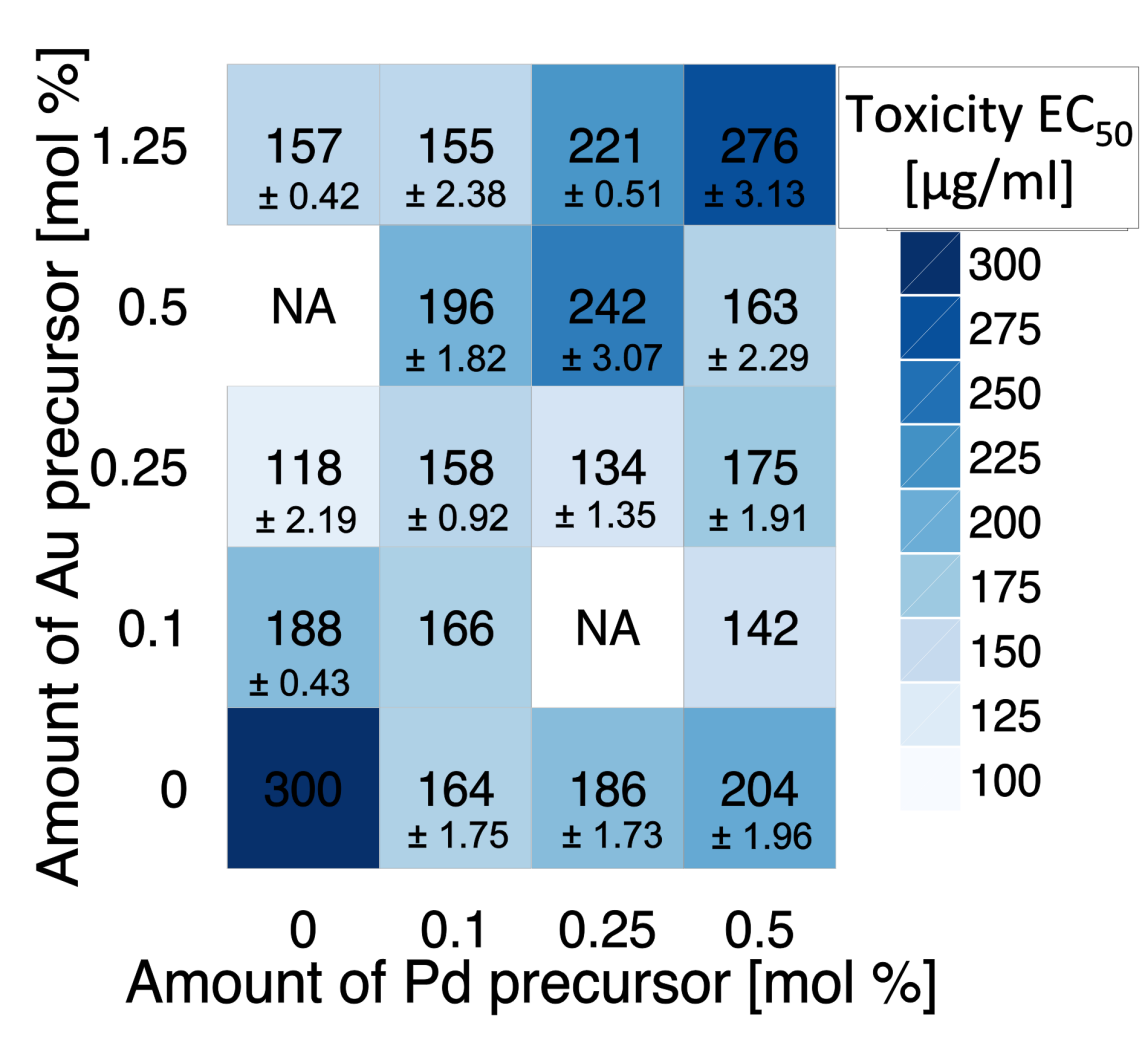
Cytotoxicity of TiO_2_ modified with Pd and/or Au nanoparticles (NA: not available, these combinations were not investigated).

The correlation between the amount of metal precursor and the structure of nanoparticles is presented in Figure 1. For lower concentrations of Au precursor (Pd > Au), core–shell types of nanoparticles (Au_core_/Pd_shell)_ were formed more likely, whereas the increasing concentration of Au (Au > Pd) resulted in the formation of alloy structures. More details about the characterization of Au/Pd-TiO_2_ structures are reported in our previous study [5]. The relationship between the amount of noble metals (Au, Pd) in the sample and the cytotoxicity to CHO-K1 cells expressed as EC_50_ values ranging from 118.39 ± 2.19 to 275.63 ± 3.13 µg/mL are presented in Figure 2.

The cytotoxic effect of monometallic Au-TiO_2_, Pd-TiO_2_ and bimetallic Au/Pd-TiO_2_ was stronger than one that was observed for pure TiO_2_ (Figure 2). Overall, the experimental results showed that TiO_2_-based nanoparticles exhibit low and moderate cytotoxicity. Several possible mechanisms of cytotoxic action can be taken into account to explain observed results. First, it is well known that ionic Au and Pd are one of the most toxic forms among the noble metals. The main factor responsible for their toxicity is related to the release of ions from the TiO_2_ surface, the generation of reactive oxygen species (ROS) and the subsequently induced oxidative stress [4,46]. For example, according to Li et al. [4] and Qiu et al. [47], the cytotoxicity of Au NPs occurs via the generation of ROS and the peroxidation of lipids. Katsumiti et al. [48] found that citrate-stabilized Au NPs exhibited a relatively low cytotoxicity, being less toxic than ionic Au, but more toxic than bulk Au. Similar results have been obtained by Contreras et al. [49]. Authors indicated that Ag^+^ is the most toxic among various metal cations (i.e., Ag^+^, Cu^+^, Cu^2+^, Co^2+^, Ni^2+^, Fe^3+^) and that the cytotoxicity of metals to human gingival fibroblast (HGF) decreases in the following order: (most toxic) AgCl > CuCl_2_ > CuCl, CoCl_2_ > NiCl_2_ > FeCl_2_, FeCl_3_ (least toxic). In addition, they have demonstrated that Ag^+^ ions can induce internucleosomal DNA fragmentation that can lead to non-apoptotic cell death [6]. Li et al. [4] demonstrated that bacterial activity of hybrid Ag-TiO_2_ materials based on Degussa P25 TiO_2_ NPs is related to the enhanced ROS generation and release of Ag^+^ ions.

### Quantitative structure–activity relationship modeling of cytotoxicity

The data summarized in the Table 1, suggest that the antimicrobial activity of the studied nanomaterials varies only insignificantly. Thus, we have chosen the cytotoxicity (EC_50_) to Chinese hamster ovary (CHO-K1) cells as a target activity for further study. Empirical variables (i.e., average size, BET surface) that quantitatively describe the features of nanoparticles structure were considered as descriptors (Table 2). It is interesting to point out, that there was no significant linear correlation between the considered cytotoxicity and the descriptors (the Pearson correlation coefficient was lower than 0.5). To address this problem and to uncover the nonlinear relationship underlying measured data the Gaussian process approach was therefore used.

**Table 2:** Summary data of developed descriptors.

sample	type of NP^a^	BET surface area (m^2^/g)	size_min_ of NPs (nm)	size_max_ of NPs (nm)

Pure TiO_2_	—	154 ± 5	0	0
0.1Au	p	168 ± 5	8	31
0.25Au	p	139 ± 5	12	63
1.25Au	p	140 ± 5	12	129
0.1Pd	p	154 ± 5	4	4.5
0.25Pd	p	182 ± 5	4	11
0.5Pd	p	139 ± 5	3	12
0.5Pd_1.25Au	a	139 ± 5	8	45
0.1Pd_0.1Au	cs	156 ± 5	6	25
0.1Pd_0.25Au	a	157 ± 5	63	140
0.1Pd_0.5Au	a	148 ± 5	54	200
0.1Pd_1.25Au	a	179 ± 5	5	17
0.5Pd_0.1Au	cs	136 ± 5	15	35
0.5Pd_0.25Au	cs	164 ± 5	19	40
0.5Pd_0.5Au	cs	153 ± 5	8	80
0.25Pd_0.25Au	cs	159 ± 5	17	170
0.25Pd_0.5Au	a	158 ± 5	7	70
0.25Pd_1.25Au	cs	145 ± 5	16	68

^a^p: pure NPs; a: alloy NPs; cs: core–shell.

The power of the Gaussian process approach, which uses lazy learning, is that it has an inherent ability to select the meaningful descriptors relevant to the endpoint of interest. In other words, the Gaussian process approach does not require a subjective selection of the model parameters (i.e., the most influential descriptors). Owing to this, variation of sizes and BET surface of nanoparticles were found to influence the EC_50_ value, and consequently were used to derive the nano-QSAR model. These findings clearly demonstrate that the cytotoxicity depends on particle size and surface area, which is in line with the recent experimental results. For instance, Coradeghini et al. [50] investigated the particle size dependent cytotoxicity of Au NPs (0.8–15 nm) to four different cell lines demonstrating that smaller NPs (1.4 nm) were more cytotoxic than bigger NPs. Previous studies also reported that a larger surface area (as a potential source of a larger number of ions or other reactive species) can contribute significantly to the higher reactivity [51–52].

The nano-QSAR model developed here is characterized by *R*^2^ = 0.94, and RMSE = 9.52 values for the training set; *R*^2^_bagging_ = 0.94 and RMSE = 37.5 for the bagging set; Q^2^_EXT_ = 0.98 and RMSEP = 9.40 for the test set. Detailed information on the experimental and predicted EC_50_ as well as residuals calculated between the actual and fitted values with the nano-QSAR model are summarized in Table 3.

**Table 3:** Observed and predicted values for cytotoxicity of TiO_2_-based NPs.

sample	status	observed cytotoxicity EC_50_ (µg/mL)	predicted cytotoxicity EC_50_ (µg/mL)	errors associated with predictions (µg/mL)

1.25Au	training (bagging)	156.80	162.38 (165.33)	5.58
0.25Pd	training	186.35	187.03	0.68
0.1Pd_0.1Au	training (bagging)	165.96	169.12 (159.26)	3.16
0.25Pd_1.25Au	training	162.68	169.91	7.23
0.5Pd_1.25Au	training (bagging)	275.63	264.60 (197.37)	11.03
0.1Pd_1.25Au	training	155.35	143.02	12.33
0.25Au	training	118.39	126.65	8.26
0.1Pd_0.5Au	training (bagging)	195.73	199.78 (171.02)	4.05
0.25Pd_0.25Au	training (bagging)	141.91	142.69 (156.06)	0.78
0.1Pd	training	164.18	163.35	0.83
0.5Pd_0.25Au	training	241.73	224.70	17.03
0.5Pd_0.1Au	training	134.28	150.21	15.93
0.25Pd_0.5Au	test	175.12	170.42	4.7
0.1Au	test	187.50	180.69	6.81
0.5Pd_0.5Au	test	220.80	210.40	10.4
0.1Pd_0.25Au	test	158.00	150.18	7.82
0.5Pd	test	204.02	193.24	10.78

**Figure 3:**
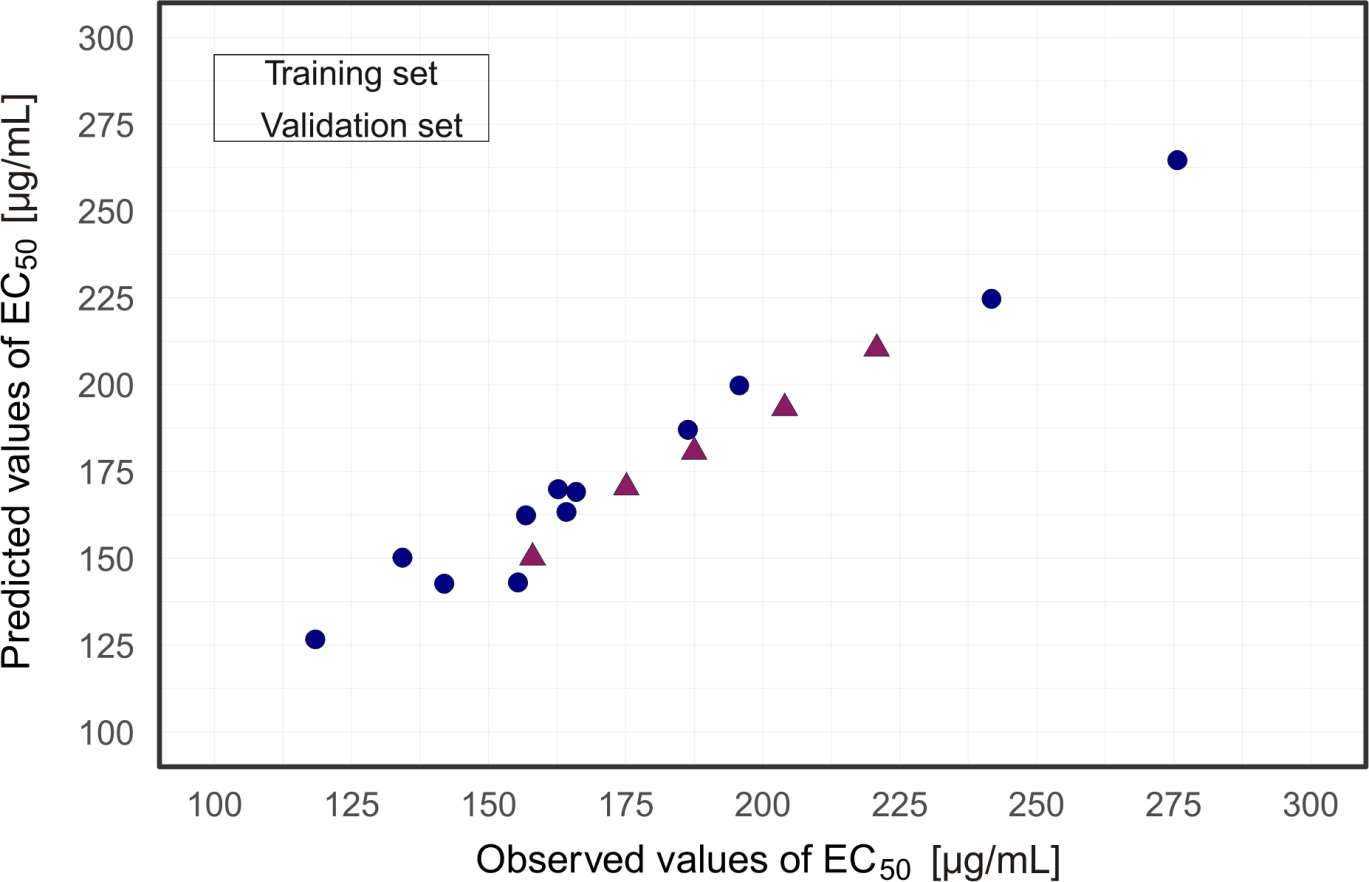
Plot of experimentally determined (observed) vs predicted values of cytotoxicity based on developed nano-QSAR model.

A plot of experimentally determined vs predicted values for the general model is presented in Figure 3. This plot revealed a good agreement between the observed and predicted values of cytotoxicity for the 17 TiO_2_-based NPs from both the test set and training set. Presented results (Figure 3) verified the predictive ability of the evolved model.

**Figure 4:**
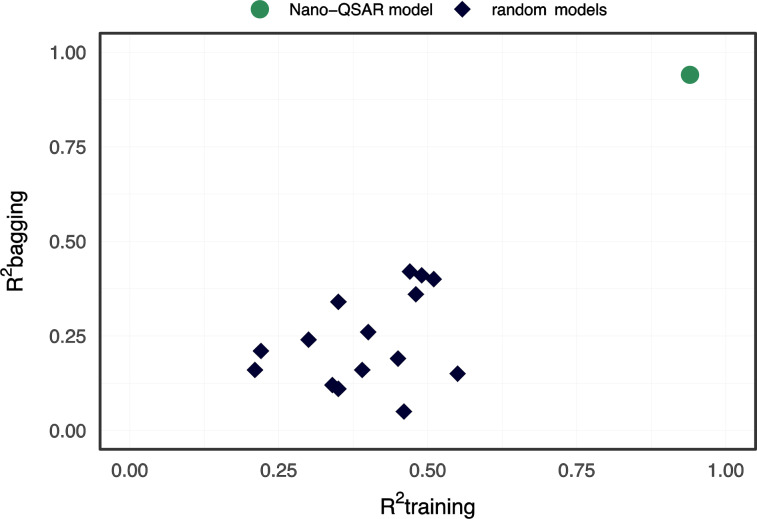
Y-randomization test.

The developed nano-QSAR model was examined through a Y-scrambling test. We found that *R*^2^ for randomization ranged from 0.21 to 0.55, while *R*^2^_bagging_ ranged from 0.05 to 0.42 (Figure 4). The RMSE values for both randomized training and bagging sets were higher than 50.

**Figure 5:**
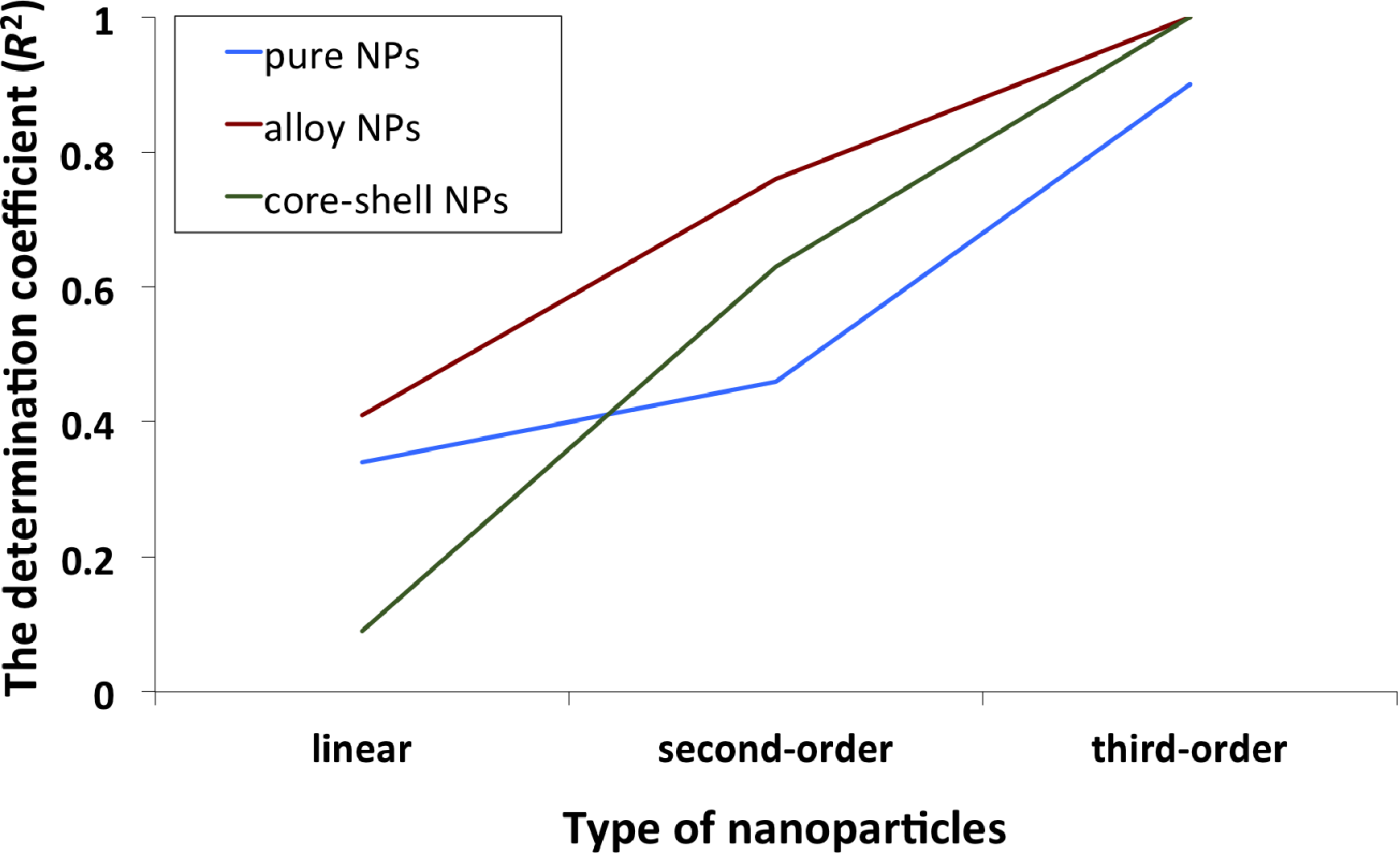
The graphical representation of goodness-of-fit and types of dependencies for the developed models.

Bearing in mind the nature of modeling nonlinear relationships, one should remember that the contribution of each descriptor to the studied biological activity cannot be individually interpreted, unlike in linear modeling. On the other hand, as we set a polynomial kernel for nano-QSAR modeling, it became possible to investigate set of polynomials separately. For this purpose, separate equations were obtained directly from the experimental data. As summarized in Table 4 and Figure 5, linear modeling failed for all types of NPs, while third-order polynomials were overfitted (R^2^ > 0.9 for five data points) for alloys and core–shell NPs.

**Table 4:** Correlations value for each type of equation.

type of NPs	Equation	equation type		
		linear	second-order polynomial	third-order polynomial

pure	1	0.34	0.46	0.90
alloy	2	0.41	0.76	1.00
core-shell	3	0.09	0.63	1.00

With regard to the results obtained, we assumed that the best equations included in the final nano-QSAR model should be the third-order correlation for cytotoxicity and average size of nanoparticles loaded with pure Pd or Au (*R*^2^ = 0.90); and second-order for alloys and core–shell systems, with *R*^2^ 0.76 and 0.62, respectively (Equations 1–3):

[1]
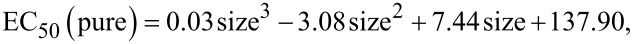


[2]
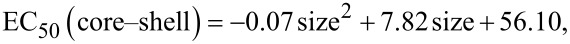


[3]
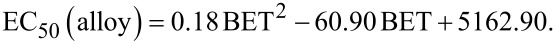


We would like to point out that the developed equations are simplified from the extracted initial Gaussian predictor. It should be highlighted, that in the case of core–shell systems, polynomial relationships were observed between cytotoxicity and average size of nanoparticles, whereas in the case of alloys, a polynomial relationship was observed between cytotoxicity and BET surface.

Using Equations 1–3 we calculated RMSE values for each type of TiO_2_-based nanoparticles, which were: 8.9 for pure NPs, 26.0 for core–shell NPs and 17.3 for alloy NPs, respectively. As one can notice, small set of separate polynomials provided cytotoxicity values with higher errors than the initially developed nano-QSAR model.

As mentioned before, one of the potential disadvantages of the Gaussian process approach may be the difficulty associated with the impossibility of direct interpretation of the selected descriptors in terms of the endpoint of interest. However, one can directly apply Equations 1–3 for predictive purposes, bearing in mind, that each equation is applicable only for a certain topology (pure, alloy, core–shell) or chemistry (Au, Pd, Au/Pd) of TiO_2_-based nanoparticles.

Taken together, these results suggest, that multimode action of nanoparticles at different Au and Pd concentrations are driven by nonlinear size-effects and surface-effect patterns. Experimental findings were supported by the developed nano-QSAR model. Ultimately, the developed model is a proof that the slightest variation of the initial experimental conditions can cause non-linear changes of nanotoxicity, so appropriate experimental conditions should be defined as good as possible before the experiment.

## Conclusion

Without a doubt, the development of computational methods that may significantly minimize the need for animal testing, and reduce the cost and time of empirical tests is essential for (eco)toxicological hazard assessment, regulatory frameworks and materials science. The intention of the present work was to demonstrate the usefulness of a quantitative structure–activity relationships approach for investigating the cytotoxic effect of surface-modified TiO_2_-based nanoparticles. In the present study, we have focused on the identification of the main physicochemical parameters that may govern toxic effects of the TiO_2_-based mono- and bimetallic nanoparticles to mammalian Chinese hamster ovary (CHO-K1) cells and to bacteria *E. coli*. The findings from this research study have clearly demonstrated that the cytotoxic effect of monometallic Au-TiO_2_, Pd-TiO_2_ and bimetallic Au/Pd-TiO_2_ was greater than the one observed for pure TiO_2_. At the same time, when MIC was determined, inhibition of bacterial growth was not observed for the investigated nanomaterials, up to the highest tested concentration. When comparing the metal content, the nanoparticles with higher concentration of palladium appear to be more cytotoxic. It is worth noting that a general understanding of the factors that play a prevailing role in the toxicity of second-generation nanoparticles is crucial to establish the mechanism of their toxicity. The overall results of this study reveal that the size and specific surface (as a potential source of a larger number of metal ions) are important factors for toxicity evaluation of modified TiO_2_-based nanoparticles. In a further perspective, more detailed investigations, including the use of a variety of transition metal-doped TiO_2_ precursors, variations of the initial experimental conditions, and/or various endpoints of environmental and human health relevance will be necessary.

## Experimental

### Materials

Titanium(IV) isopropoxide (TIP, 97%), palladium(II) chloride (5 wt % in 10 wt % HCl) and HAuCl_4_ (Au ≈ 52%) were purchased from Sigma-Aldrich. Cyclohexane, isopropyl alcohol, hydrazine, acetone, AOT (dioctylsulfosuccinate sodium salt) obtained from POCH S.A. (Poland) were used without further purification. F12 medium, streptomycin and penicillin, glutamine, and heat-inactivated fetal bovine serum (FBS Hi) were purchased from Gibco^®^ Life Technologies. Microbiological media (tryptic soy broth (TSB) and tryptic soy agar (TSA)) were purchased from Becton Dickinson and Company. WST-8 reagent [2-(2-methoxy-4-nitrophenyl)-3-(4-nitrophenyl)-5-(2,4-disulfophenyl)-2*H* tetrazolium, monosodium salt] was obtained from Wako (Osaka, Japan).

### Synthesis of Au/Pd-TiO_2_ nanoparticles

Titania (TiO_2_) modified with nanoparticles was obtained by hydrolysis of TIP in a water/AOT/cyclohexane microemulsion containing Au and Pd precursors in water cores. Mixing was carried out for 1 h under nitrogen; Au and Pd were then reduced by dropwise addition of a microemulsion containing the reducing agent (hydrazine). Titanium isopropoxide was added into the microemulsion system containing Au and Pd nanoparticles. The microemulsions were mixed and purged with nitrogen for 24 h and the obtained precipitate was washed, dried and calcined for 3 h at different temperatures, as described previously [53].

### Toxicity to Chinese hamster ovary (CHO-K1) cells

Nanoparticles were ground for 5 min using a mortar and pestle, suspended to a concentration of 1 mg/mL in complete cell culture medium with 0.1% pluronic F68 (cytotoxicity assay) or TSB (MIC determination) and sonicated in a water bath for 30 min at 37 °C. Cytotoxicity was determined using the Chinese hamster ovary cell line (CHO-K1) (ATCC^®^ CCL-61^™^). The sensitivity of three different cell lines: CHO-K1 and two human lung (cancer and normal) cell lines (A549, BEAS-2B) to the tested nanomaterials was studied in a preliminary experiment. CHO-K1 proved to be the most sensitive, allowed for the determination of EC_50_ values for all tested nanomaterials and therefore was selected for the main experiment. A colorimetric assay with WST-8 reagent was used for the cell viability tests: CHO-K1 cells were pre-cultured in F12 culture medium supplemented with 2 mM L-glutamine, 1% penicillin/streptomycin solution, and 10% heat-inactivated fetal bovine serum (FBS) at an initial density of 1 × 10^5^ cells/mL in 24-well plates. Cells were exposed to nine different concentrations of nanoparticles (from 1.56 to 300 µg/mL) for 24 h. Because Au nanoparticles absorb light in the visible region, the plates were centrifuged to avoid interference with the assay. At the next step, 100 µL of medium from each cell culture was transferred to a 96-well plate and the absorbance at 450 nm was measured. Cell viability was calculated as means of three independent experiments and expressed as the percentage of the viability of exposed cells vs controls. Concentration–response curves were fitted using the nonlinear least-squares method. Calculations were carried out with the R environment (http://www.r-project.org).

### Antibacterial activity

The antibacterial activity of studied nanomaterials was evaluated using Gram-negative bacteria *Escherichia coli* (NBRC 3972, NITE Biological Resource Center). Two methods were employed: MIC (minimal inhibitory concentration) determination and agar diffusion test.

### Minimal inhibitory concentration (MIC)

MIC was determined by the serial twofold dilution microtiter plate method in TSB. Wells containing serially diluted compounds and compound-free controls were inoculated with an overnight culture of bacteria to a final concentration of 5 × 10^5^ cfu/mL. The plates were then incubated for 24 h at 37 °C. The microbial growth was quantified in each well by measuring the optical density at λ = 580 nm. MIC was defined as the concentration of the compound, which resulted in at least an 80% decrease in turbidity relative to that of the compound-free growth control well.

### Agar diffusion test

Approximately 20 mL of sterile molten TSA was poured into sterile Petri plates. The solid medium was inoculated with 200 µL of an overnight culture (density of 10^6^ cfu/mL) of bacteria. Nanomaterials were suspended in TSB to a concentration of 4 mg/mL. Then, 20 µL of this suspension was dispensed onto a disc placed on the agar medium surface (diameter of each disc: ca. 6mm, three discs on each plate). The plates were incubated for 24 h at 37 °C. Diameters of growth inhibition zones were measured.

### Quantitative structure–activity relationships modeling

The QSAR approach is based on the assumption that the variance in molecular structures, encoded by numerical parameters (so-called “descriptors”), correlates (using statistical approaches) with the variance in biological activity. We used as descriptors the measured experimental parameters (Table 2). For the purposes of statistical modeling, Gaussian process predictor was applied [54]. Gaussian process predictor is a probabilistic approach to learning in kernel machines. A Gaussian process is a generalization of the Gaussian probability distribution [54]. The initial dataset was split between training and test sets on the basis of randomly method (OECD, 2014). The training set covers ca. 80% of the initial dataset; the test set covers the remaining ca. 20%. Descriptors from training set were standardized and then the Gaussian process using normalized polynomial kernel was applied. The statistical quality of the QSAR model and its predictive ability were assessed using determination coefficient (*R*^2^) for training set, bagging validation coefficient (*R*^2^_bagging_) for bagging set, external validation coefficient (*Q*^2^_EXT_), and root-mean-square errors of calibration for the training set (RMSEC), and root-mean-square errors of validation (RMSEP) for the test set (see Equation 4-7) [55–58].

[4]
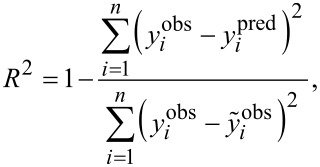


[5]
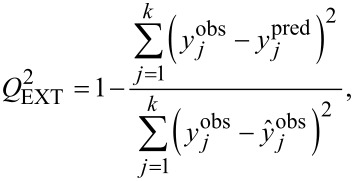


[6]
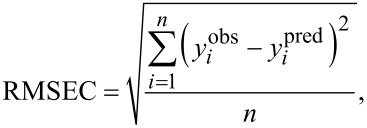


[7]
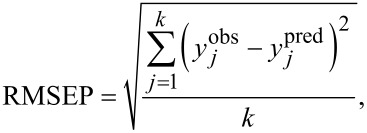


where y*_i_*^obs^ is the experimental (observed) value of the property for the *i*-th compound from the training set; *y_i_*^pred^ is the predicted value for the *i*-th compound from the training set; 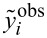 is the mean experimental value of the property in the training set; y*_j_*^obs^ is the experimental (observed) value of the property for the *j*-th compound from the validation set; y*_j_*^pred^ is the predicted value for *j*-th compound from the validation set; 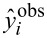 is the mean experimental value of the property in the validation set; *n* is the number of compounds in the training set; *k* is the number of compounds in the validation set; and *R*^2^_bagging_ is the average value of *R*^2^ of each model.

Finally, the developed Nano-QSAR model was checked for chance correlation and its robustness was examined through a Y-scrambling test (so-called dependent-variable scrambling test) [58].
